# Antigenic Maps of Influenza A(H3N2) Produced With Human Antisera Obtained After Primary Infection

**DOI:** 10.1093/infdis/jiv367

**Published:** 2015-07-03

**Authors:** Judith M. Fonville, Pieter L. A. Fraaij, Gerrie de Mutsert, Samuel H. Wilks, Ruud van Beek, Ron A. M. Fouchier, Guus F. Rimmelzwaan

**Affiliations:** 1 Centre for Pathogen Evolution, Department of Zoology, University of Cambridge; 2 WHO Collaborating Centre for Modelling, Evolution, and Control of Emerging Infectious Diseases, Cambridge, United Kingdom; 3 Department of Viroscience, Erasmus MC; 4 Department of Pediatrics, Erasmus MC–Sophia, Rotterdam, The Netherlands

**Keywords:** primary infection, influenza, human antisera, antigenic cartography, antibody landscapes

## Abstract

**Background:**

Antigenic characterization of influenza viruses is typically based on hemagglutination inhibition (HI) assay data for viral isolates tested against strain-specific postinfection ferret antisera. Here, similar virus characterizations were performed using serological data from humans with primary influenza A(H3N2) infection.

**Methods:**

We screened sera collected between 1995 and 2011 from children between 9 and 24 months of age for influenza virus antibodies, performed HI tests for the positive sera against 23 influenza viruses isolated between 1989 and 2011, and measured HI titers of antisera against influenza A(H3N2) from 24 ferrets against the same panel of viruses.

**Results:**

Of the 17 positive human sera, 6 had a high response, showing HI patterns that would be expected from primary infection antisera, while 11 sera had lower, more dispersed patterns of reactivity that are not easily explained. The antigenic map based on the high-response human HI data was similar to the map created using ferret data.

**Conclusions:**

Although the overall structure of the ferret and human antigenic maps is similar, local differences in virus positions indicate that the human and ferret immune system might see antigenic properties of viruses differently. Further studies are needed to establish the degree of similarity between serological patterns in ferret and human data.

Influenza viruses are notorious for their continuous antigenic evolution and resulting ability to escape prior immunity and reinfect previously exposed hosts [1, 2]. To protect against influenza virus infection, an effective vaccine is available, but to maintain vaccine effectiveness, the antigenic properties of circulating viruses need to be known, and the vaccine strains have to be updated when appropriate to avoid antigenic mismatch [3, 4]. To this end, the World Health Organization (WHO) coordinates a global influenza surveillance network that routinely characterizes the antigenic properties of isolated viruses, using the hemagglutination inhibition (HI) assay [5]. For antigenic characterization, sera from a panel of ferrets that were each experimentally infected with a different virus strain are used. The resulting HI tables contain serum antibody titers with patterns that are now fairly readily interpretable and understood, because antigenic differences among virus strains can be mapped with antigenic cartography [1]. An antigenic map quantifies and displays antigenic differences as distances between viruses, such that similar viruses cluster closely on the map, whereas viruses that are antigenically different are found further away. This method is now routinely used to assist the vaccine strain selection process and surveillance activities of the WHO and is based on large HI tables of influenza virus isolates that were titrated against a panel of ferret sera.

Although human sera are used in the vaccine strain selection process for activities such as evaluating the response to vaccines in vaccine trials, their analysis is too complicated for trivial use in the antigenic characterization of influenza viruses. For example, when a human has had 2 separate exposures to influenza virus, titers to both infecting viruses will be increased, which may potentially be interpreted incorrectly as an antigenic similarity between the 2 different infecting viruses. A recent solution to the interpretation of human serological data is the generation of an antibody landscape, which expands an antigenic map in another dimension by displaying the HI titers measured for the human serum for each virus [6].

In addition to the fact that human data are typically representative of multiple exposures, there are manifold other reasons, often involving factors associated with experimental control, why ferret data are preferred for the antigenic characterization of virus isolates: the exact infecting virus strain for a ferret is always known, whereas for human infections this is much less common; ferret serum samples can be obtained at a standardized time point after infection, when there is a peak in antibody titers, increasing the sensitivity of interpretations of the resulting HI tables; sufficiently large serum volumes for extensive and repeated testing are available; and sera can be newly generated against viruses that circulated in the past.

The traditional interpretation of ferret HI data when performing antigenic characterization, the WHO vaccine strain selection process, and the expansion of a ferret-based antigenic map into an antibody landscape all implicitly assume that antigenic characteristics are similar to what would be measured in a human, when using the ferret animal model. Interestingly, a small comparison of titers from swine sera and ferret sera gave similar results for swine influenza A(H3N2) strains [7], and results of antigenic characterization of avian influenza A(H5N1) isolates with ferret sera were consistent with titer patterns in HI tests with avian antisera [8, 9]. Similarly, it is important to carefully compare and know the antigenic properties of human viruses in relation to the human immune system and antibody response. To our knowledge, validation studies to confirm the use of the ferret as a model system for the antigenic characterization of seasonal influenza virus isolates in the context of different human immune responses have not been performed. This is understandable, because it is challenging to obtain human sera that fulfill the stringent requirements of having been collected after the individual's first influenza virus infection and during the circulation of different antigenic clusters.

This study presents the first human antigenic map made for influenza viruses and is based on a data set of historical and recent serum samples, to enable antigenic characterization of various influenza A(H3N2) isolates recognized by human antibody repertoires. By having access not only to recent but also to historical serum samples from children, we were not limited to the characterization of a small subset of recently circulating viruses. Instead, we investigated serum responses against viruses isolated over 22 years of influenza A(H3N2) evolution, across 9 antigenic clusters. These data enable a comparison of antigenic properties of viruses defined by human sera after natural infection to those defined by ferret sera obtained after experimental infection.

## METHODS

Human serum samples were selected from the serum bank of the Department of Viroscience at the Erasmus Medical Center (Rotterdam, the Netherlands). Only sera from patients seen at the Sophia Hospital Pediatrics ward who, and of whom the caregivers, did not object to scientific use of excess material were included in this study. The study protocol was reviewed and approved by the medical ethics board of the Erasmus University Medical Center (study number MEC-2012-181). Informed consent was waived because patient inclusion was performed retrospectively and data handled anonymously. From the 5129 individuals, we selected individuals aged 270–729 days, to minimize interference from transplacentally acquired maternal immunoglobulin G antibodies (immunoglobulin A antibodies in breast milk were not considered relevant for our studies of sera) and multiple seasonal influenza virus infections [10, 11]. We excluded sera from patients in whom the possibility of nonnaturally obtained antibody response existed, and samples from patients with immune deficiencies were also excluded. Based on surveillance data of the incidence of influenza A(H3N2) infection in the Netherlands, we selected samples that were collected within 2 weeks either side of the epidemic season window. We then selected samples with at least 150 µL of serum, leading to a final sample set of 72 sera (Supplementary Methods).

HI assays were used to screen the serum samples for influenza virus–specific antibodies against the vaccine strain for the antigenic cluster that circulated in that season (Supplementary Data Set 1), using the standard protocol [12, 13]. The 17 influenza virus–positive samples were subsequently tested further with the HI assay against a panel of 23 viruses (Supplementary Data Set 2). Supplementary Table 1 lists the frequencies for the various reasons for hospitalization and diagnostic testing of these 17 patients, of whom 4 had underlying medical conditions (congenital kidney disease, bone formation disorder, neonatal cataract, and recurrent wheezing). For comparative purposes, we tested the sera of 24 ferrets against the same panel of viruses with the HI assay. Supplementary Data Sets 3–7 show the HI results and subsets used for antigenic cartography (Supplementary Methods).

Antigenic maps were used to infer antigenic differences among virus strains, with each titer in the HI table specifying a target distance for the virus and serum points in the antigenic map [1].

Antibody landscapes were constructed as described by Fonville et al [6], except for a modification to model the effects of the *x* and *y* antigenic coordinate variables independently (Supplementary Methods).

## RESULTS

We selected sera from a biobank of stored blood specimens collected from children between the ages of 9 and 24 months for any viral diagnostic analysis between 1995 and 2012. Upon screening the 72 selected sera against influenza virus–specific antibodies with the HI assay, 17 (23.6%) had an HI titer of ≥10 against the screening virus. Interestingly, there was a dichotomy in the HI screening titers, with one group of relatively high-responding individuals (6 [8.3%]; HI titer range against screening virus, 240–3840), and a group of low responders (11; HI titer range against screening virus, 10–60; Supplementary Data Set 2).

**Figure 1.**
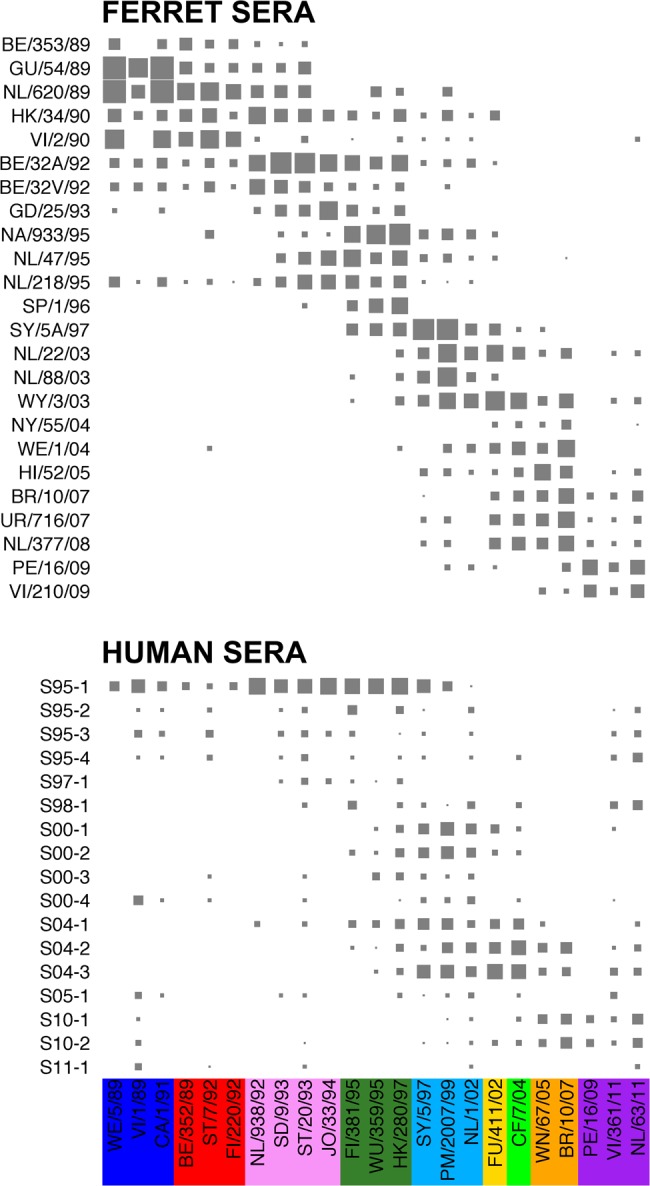
The measured hemagglutination inhibition (HI) titer with an increasing symbol size (<10 are size 0) for the ferret sera (top) and human sera (bottom), sorted by the year of isolation of the infecting virus and the year of serum collection, respectively, against the viruses sorted by their antigenic cluster. Different antigenic clusters are indicated with different colors, as follows: blue, Sichuan-87 cluster; red, Beijing-89 cluster; pink, Beijing-92 cluster; dark green, Wuhan-95 cluster; light blue, Sydney-97 cluster; yellow, Fuijan-02 cluster; light green, California-04 cluster; orange, Brisbane-07 cluster; and purple, Perth-09 cluster. See also Supplementary Data Sets 2 and 3.

The 17 influenza virus–positive human sera and 24 ferret sera were subsequently titrated with HI against a panel of viruses. Figure 1 shows the resulting HI titer patterns, with viruses color-coded by antigenic cluster, as determined from previously published antigenic maps based on ferret sera [1, 6]. Evolution of influenza A(H3N2) virus since its introduction in humans in 1968 has included the addition of glycosylation sites [14, 15]. Although the shielding of epitopes by glycans has been reported frequently to alter or prevent the ability of neutralizing antibodies to bind the virus by masking or modifying antigenic sites [16, 17], we do not see significant changes in the number of antigenic clusters recognized by sera over time, in line with the lack of synchronicity between antigenic cluster transitions and changes in glycosylation [18].

Figure 1 highlights how titers between sera and viruses that are many years apart (long-distance titers) are more common for the human sera than for the ferret sera: for example, sera obtained after primary infection from the 3 low responders in the 1994–1995 season (S95-2, S95-3, and S95-4) had titers to strains isolated in 2011, whereas such patterns were uncommon in the ferret data. Similarly, S10-2 and S11-1 had titers to strains that circulated around 2 decades before the child was born. Although some serum-virus combinations in the ferret data had titers when they would not be expected, this is a more common feature in the human data, particularly in the sera of low responders. In general, the ferret sera appear to show stronger and narrower patterns of reactivity than the human sera.

**Figure 2.**
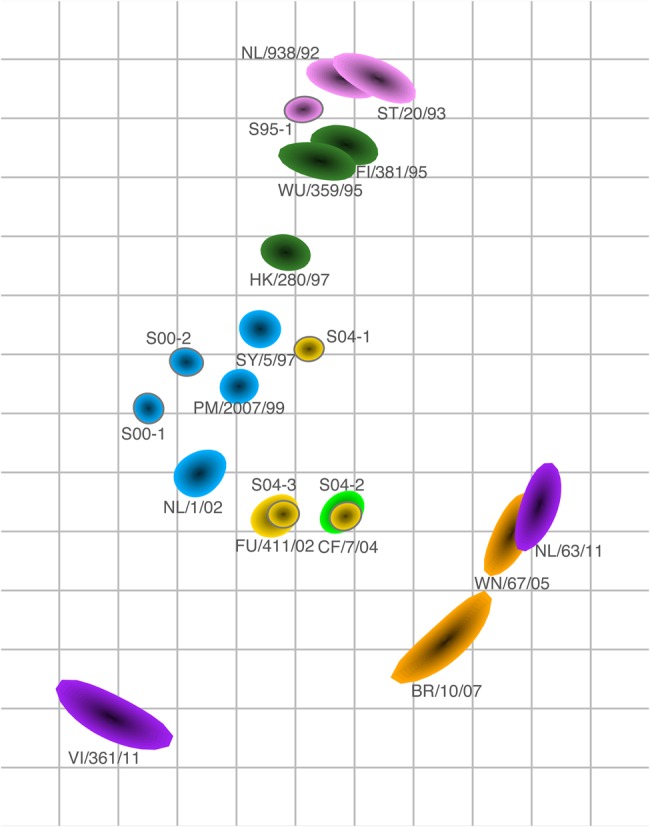
Antigenic map based on the hemagglutination inhibition (HI) titers of the sera of the 6 high responders and representative virus isolates of different antigenic clusters [1]. In an antigenic map, both vertical and horizontal axes represent antigenic distance. The spacing between grid lines is 1 antigenic unit distance, corresponding to a 2-fold dilution in the HI assay (eg, 2 units correspond to a 4-fold dilution, and 3 units correspond to an 8-fold dilution). Different antigenic clusters are indicated with different colors as in Figure 1. Sera are shown with a gray outline, and serum names indicate the season of serum collection (eg, specimen S95-1 was collected in 1994–1995). The size and shape of each symbol reflects the certainty in positioning of the virus or serum and represents the coordination confidence area as locations on the map that the point could also occupy without increasing the stress of the map with >0.5. The shape size typically increases as fewer HI titers are available. This map was made without a minimum column basis.

We proceeded by focusing our analyses on the sera of the high responders and generated an antigenic map based on sera from the following 6 individuals: S95-1, S00-1, S00-2, S04-1, S04-2, and S04-3 (Supplementary Data Set 4). The number of viruses per antigenic cluster can vary and still result in robust antigenic maps, as long as the density of viruses is high enough. Viruses can be placed reliably on a map once there are numeric titers (ie, titers that are not <10) against at least 2 sera, and we mapped the eligible 14 viruses, isolated between 1992 and 2011. Figure 2 displays the resulting antigenic map, with viruses colored by antigenic cluster and antisera colored by the antigenic cluster that was present in the season the sample was obtained. Interestingly, the viruses mostly still clustered by color; that is, the assignment to antigenic clusters based on ferret sera reactivity matches fairly well with the human antigenic map. The antigenic map represents the measured HI data well, as characterized by small error lines (Supplementary Figure 1).

The human sera were relatively close to the viruses for the corresponding antigenic cluster, with the possible exceptions of sera S04-1 and S04-2. S04-1 may have been infected before the 2003–2004 season, in the 2001–2002 season (although high levels of maternal antibodies would be expected for an individual this soon after birth), or in the 2002–2003 season, when the relatively small influenza epidemic in the Netherlands was dominated by strains that were antigenically like the Sydney-97 antigenic cluster [19]. An infection with a Sydney-97–like virus would explain the location of this serum in the antigenic map close to such isolates. The S04-2 serum was antigenically slightly more advanced than the surveillance data for the 2003–2004 influenza season in the Netherlands [20] and similar to the California-04–like viruses that circulated during the 2004–2005 epidemic [21]. Supplementary Figure 2 shows a similar antigenic map of the children's HI data, made with a column basis of at least 1280 (which is conventionally used for the analysis of ferret serology), and underlines how the antigenic relationships and positions of the sera were similar for both analyses.

**Figure 3.**
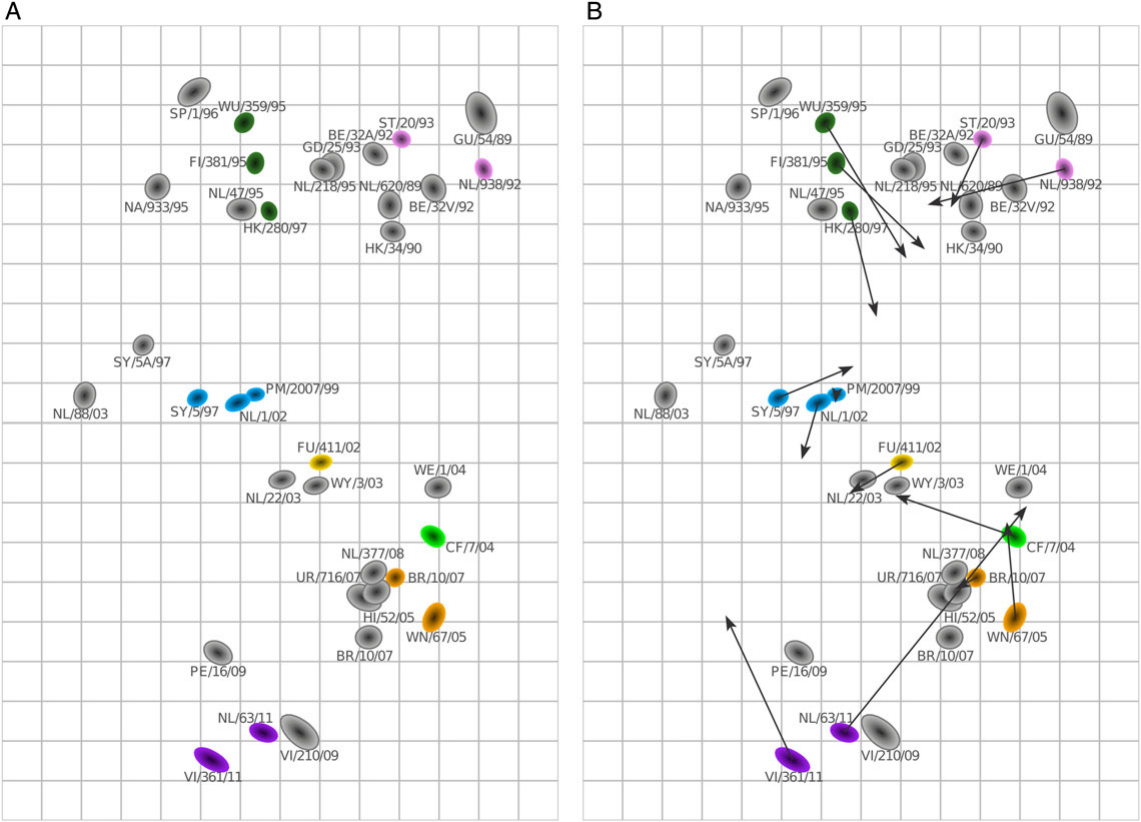
Comparison of antigenic maps based on ferret and human sera. *A*, Antigenic map based on 21 ferret sera, with the same virus set as in Figure 2 (made without minimum column basis). Sera are shown in gray. *B*, Procrustes arrows from each virus point to its corresponding position in the human map in Figure 2.

Importantly, we can compare the positioning of the viruses on the antigenic map as defined by the human sera with an antigenic map based on ferret sera. Figure 3*A* shows an antigenic map of 21 ferret sera (Supplementary Data Set 5) measured against the same subset of viruses, and Figure 3*B* displays procrustes arrows, in which the arrowhead indicates the corresponding positions of the viruses in the human map. Although the overall structure of the 2 maps remained similar in terms of the relative ordering of antigenic properties (eg, the Beijing-1992 and Wuhan-1995 clusters are closer to each other than to the California-2004 cluster), there are visible differences between the 2 maps. Additionally, the overall spread of the antigenic map comprises fewer antigenic units when based on human sera than when based on ferret sera. Similar observations were made when comparing the human and ferret maps made with the minimum column basis requirement (Supplementary Figure 3) and when making the ferret map based on a subset of 6 sera only (Supplementary Figure 4).

**Figure 4.**
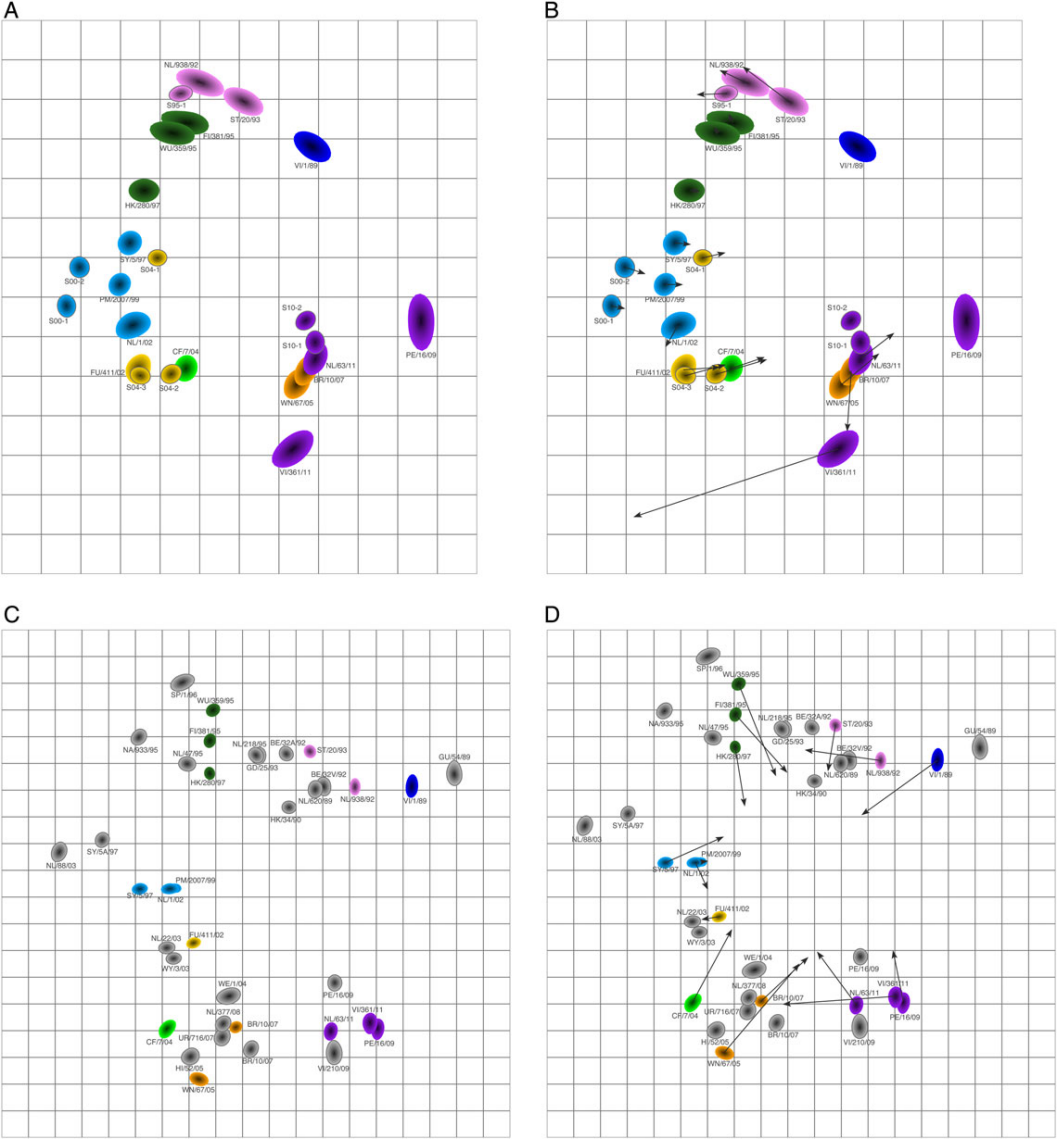
Addition of 2 additional sera to the human antigenic map. *A*, Antigenic map of sera from the 6 high responders plus 2 additional sera with titers of at least 240. *B*, Procrustes arrows displaying for each virus and serum in panel *A* its corresponding position in Figure 2. *C*, Antigenic map of ferret data, using the same virus set as in panel *A. D*, Procrustes arrows showing the position of each virus in the ferret antigenic map shown in panel *C* in the human antigenic map shown in panel *A*.

Subsequently, we explored how the human antigenic map would change when 2 of the low responders were included (these sera had at least 1 titer of at least 240, instead of a screening titer of at least 240; Supplementary Data Set 6). The resulting map, shown in Figure 4*A*, is quite comparable to the antigenic map made without these 2 sera: the procrustes arrows in Figure 4*B* are relatively small, with the exception of a large change in the position of virus VI/361/11. The new addition of viruses VI/1/89 and PE/16/09 caused the appearance of the map to become circular, as a result of some long-distance cross-reactivity of some of the recent sera against VI/1/89, pulling this virus inward. The ferret map made with the same subset of viruses (Figure 4*C* and Supplementary Data Set 7) also has a moderately circular appearance but is spaced more widely, compared with the human map (see the procrustes arrows in Figure 4*D*). In general, broader reactivity will typically result in antigenic maps becoming more circular than linear.

**Figure 5.**
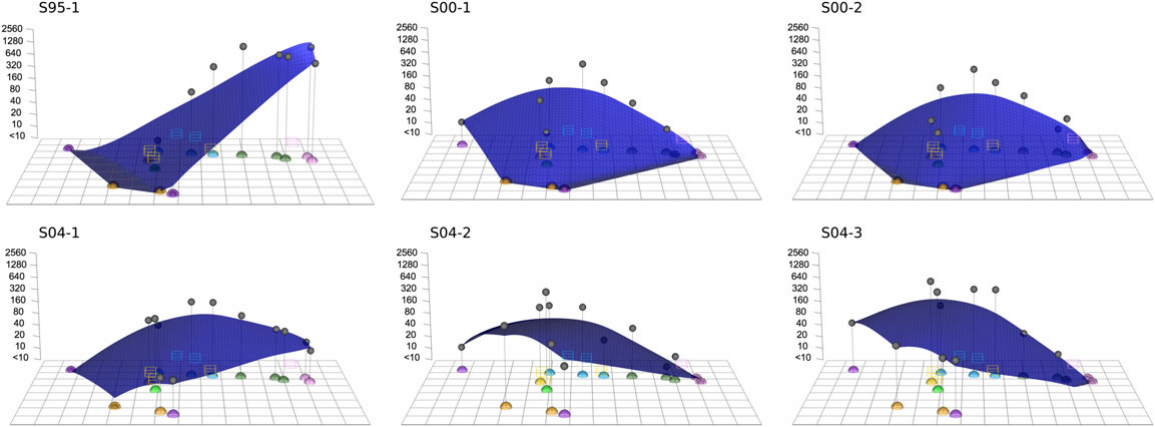
Antibody landscapes [6] of the 6 high-responding human sera using the human antigenic map of Figure 2. Antibody titers of serum specimens (gray dots) are displayed along the *z*-axis, with higher titers and higher regions of the antibody landscape corresponding to high antibody levels in an individual.

To visualize the titer pattern of an individual's serum, we added an extra dimension to the antigenic map to display the measured HI titers. By plotting a smooth surface through these titers, an antibody landscape is created, which plots the antibody recognition patterns as a function of the antigenic relationships among viruses [6]. Figure 5 displays the antibody landscapes of the 6 sera from the high responders, in which each landscape represents an individual's antibody profile, with elevations corresponding to regions in the antigenic map with higher antibody levels and depressions corresponding to regions with lower antibody reactivity. These antibody landscapes are the first ones made for human sera collected after primary infection and the first ones made on a human-serum based antigenic map. When comparing the antibody landscapes of the human sera with the landscapes of the most similar ferret sera, the landscape shapes are remarkably similar (Supplementary Figure 5).

## DISCUSSION

This study evaluated the HI titer patterns of 17 sera from children between 9 and 24 months of age. HI titers after primary infection in the literature vary widely and are known to be dependent on the interval between infection and serum collection [22–26] but also possibly on the virus subtype [22, 27] and the age of the individual [28, 29]. Some studies in the literature follow individuals from birth [24, 30] and hence have certainty about an infection being a primary exposure, while other relevant studies report titers for age groups that are typically associated with first infection [10, 22, 28, 31–35].

In the 2 studies that reported titers after primary infection with influenza A(H3N2), Wright et al observed a geometric mean titer (GMT) of 271 among 8 children between 6 and 23 months old [22], and Burlington et al found a GMT of 147 among 10 children between 1 and 4 years of age who were followed from birth [30]. In the Pienter study [10], children aged 12–24 months had a GMT against influenza A(H3N2) of 329. These values are in line with the high titers we find in the sera of high-responders.

The reason for the titer patterns observed in the low-responding individuals remains unclear. Although the timing of the sample collection in relation to any influenza virus infection is unknown and will vary from person to person, there was no difference between the low-responder and high-responder groups with respect to the time of sample collection in relation to the dates of that season's epidemic. The sera from the low responders did not have the same reactivity patterns as specimens from the high responders at lower HI values but instead showed more-dispersed reactivity patterns. Subclinical infection or antigen exposure, which would potentially lead to lower HI titers [36], do not explain these odd reactivity patterns. Sometimes these titers extended to antigenic clusters that circulated long before the child was born or to future antigenic clusters that circulated many years after collection of the serum specimen. Maternal antibodies might be able to explain the former but not the latter observation. Moreover, we had a fairly conservative selection criterion (age, >9 months) to avoid detection of maternal antibodies as much as possible, and the age ranges were similar in the low-responder and high-responder groups. The dispersed reactivity patterns could possibly be the result of the existence of natural antibodies. Alternatively, these patterns might also arise through cross-reactivity with antibodies induced after infections with other pathogens, such as other influenza viruses or even pathogens other than influenza virus, which is likely in children of this age group but not in specific-pathogen-free ferrets. Further studies are necessary to investigate the cause of the patterns seen in the low responders and will require the collection of a larger serum volume for testing.

We have, for the first time, constructed an antigenic map of influenza viruses based on human antibody recognition data. This antigenic map was based on an extensive set of human HI titers, spanning both a long interval of serum sampling and comprising 2 decades of evolution in the influenza virus test strains. Our data, and in particular the antibody landscapes, also illustrate the antibody response in terms of strength and breadth among children after their first infection and across a range of different infecting strains and seasons. It would be very interesting to study how this response changes as an individual experiences their second and third infection, to investigate how the antibody landscape of an individual is built up over time [6]. Indeed, in the context of vaccine strain selection, a previous exposure history might alter the antibody landscape and thus the immune response in a such way that data from antigenic maps based on antisera obtained after primary infection may not provide the best possible protection against influenza viruses for nonnaive individuals [6, 37, 38].

The human antigenic map was based on 6 high-responding human sera and is therefore by definition less robust than the ferret map based on 21 ferret sera. For example, the larger symbol sizes in Figure 2 for the more-recently isolated viruses indicate relatively larger uncertainty in the positioning of these viruses and are a natural result of fewer and lower numeric titers against those strains, because the sera were only from older influenza seasons. When including more-recently collected sera, as in Figure 4, the uncertainty in the positioning of the more recent viruses therefore decreased. However, even when making a map based on 6 ferret sera only (Supplementary Figure 4), the ferret map spanned a larger antigenic range than the human antigenic map. This may be because inoculation protocols and serum collection for ferrets have been optimized to yield high titers.

The human sera obtained after primary infection were similar but not identical to the ferret sera, which were obtained under controlled conditions. For example, the time of sample collection of ferret sera was standardized at 2 weeks after infection, whereas the time between infection and sample collection is unknown for the human sera. We tried to minimize variation in the times after infection at which the human sera were collected by only selecting samples obtained during a narrow window around the epidemic in the Netherlands for each season, but our findings are still limited by the absence of information on infection history. Additionally, whereas the infecting strain was known for ferret sera, this information was not available for the human sera, and some children may have been infected in a season preceding the season during which the serum was drawn. Another potential difference between the ferret and children sera is that the route and dose of infection were standardized and known for the ferrets but might have varied for the children; similarly, the ferrets were infected with laboratory-passaged viruses, whereas the children were exposed to unpassaged circulating virus. Finally, the immunological maturity between the young adult ferrets and the 9–24-month-old children might have been different.

Despite these differences between the experimentally obtained ferret sera and human sera obtained under less-controlled conditions, the ferret and human maps are globally similar, indicating that the ferret sera are able to detect useful information on overall antigenic differences among virus strains. However, the narrower spacing of the human map as compared to the ferret antigenic map, as well as the differences in the local positioning of the viruses between the maps, may be relevant. Therefore, a carefully planned prospective experiment in which a larger number of human sera are obtained at known times after infection is warranted to further test these differences. The germ-line B-cell receptor repertoires may be different between humans and ferrets, which would lead to different antibody mixtures. If the human and ferret antibodies respond to different epitopes on the hemagglutinin, antigenic maps would be different as a result. In case the different positioning is truly caused by differences in how antigenic properties are detected by ferret versus human immune systems and is relevant to vaccine strain selection, it might be helpful to supplement the ferret data with data on human sera obtained after primary infection.

## Supplementary Data

Supplementary materials are available at http://jid.oxfordjournals.org. Consisting of data provided by the author to benefit the reader, the posted materials are not copyedited and are the sole responsibility of the author, so questions or comments should be addressed to the author.

Supplementary Data
