# Supplementary material for: Antigenic Maps of Influenza A(H3N2) Produced With Human Antisera Obtained After Primary Infection
Source: J Infect Dis. 2015 Jul 3;213(1):31–8. doi: 10.1093/infdis/jiv367 (PMC4676547; doi:10.1093/infdis/jiv367)
Supplement: Supplementary Data [file supp_213_1_31__index.html]

Antigenic Maps of Influenza A(H3N2) Produced With Human Antisera Obtained After Primary Infection — Antigenic Maps of Influenza A(H3N2) Produced With Human Antisera Obtained After Primary Infection — Supplementary Data 

# Antigenic Maps of Influenza A(H3N2) Produced With Human Antisera Obtained After Primary Infection

## Supplementary Data

Supplementary Data

- Supplementary Data - Pdf file
- Supplementary Tables - xlsx file
- Supplementary Figures - pdf file
