# Supplementary material for: Antigenic Maps of Influenza A(H3N2) Produced With Human Antisera Obtained After Primary Infection
Source: J Infect Dis. 2015 Jul 3;213(1):31–8. doi: 10.1093/infdis/jiv367 (PMC4676547; doi:10.1093/infdis/jiv367)
Supplement: Supplementary Data [file supp_jiv367_jiv367supp.pdf]

SUPPORTING INFORMATION FOR:

Antigenic maps of influenza A/H3N2 virus produced with human antisera obtained after primary infection

Judith M. Fonville*[1,2,3], Pieter L. A. Fraaij[3,4], Gerrie de Mutsert[3], Samuel. H. Wilks[1,2], Ruud van Beek[3], Ron A. M. Fouchier[3], Guus F. Rimmelzwaan[3].

1 Centre for Pathogen Evolution, Department of Zoology, University of Cambridge, Cambridge CB2 3EJ, United Kingdom.

2 WHO Collaborating Center for Modeling, Evolution, and Control of Emerging Infectious Diseases, Cambridge, United Kingdom.

3 Department of Viroscience, Erasmus MC, 3015 CE Rotterdam, The Netherlands.

4 Department of Pediatrics, Erasmus MC-Sophia, 3015 CN Rotterdam, The Netherlands.

Supporting Materials and Methods:

Sample selection

Human serum samples were selected from the serum bank of the Department of Viroscience at the Erasmus Medical Centre (Rotterdam, NL). Only sera of patients seen at the Sophia Hospital Pediatrics ward who, and of whom the caregivers, did not object to scientific use of excess material were included in this study. The study protocol was reviewed and approved by the medical ethics board of the Erasmus University Medical Center (study number MEC-2012-181). Informed consent was waived because patient inclusion was performed retrospectively and data handled anonymously. From the 5129 individuals, we excluded those who were seen in the departments of the Erasmus MC that are not part of the Erasmus MC – Sophia Children's Hospital, and also only selected individuals of 270-729 days old (i.e. between 9 months and 2 years), leaving 1684 samples. Sera from patients in whom the possibility of non-naturally obtained antibody response existed (e.g. vaccination, intravenous immunoglobulin administration), or samples from patients with immune deficiencies (e.g. auto-immune disease, HIV-positive status, use of immunosuppressive medication) were excluded from use in this study. The age criterion of 9-24 months old was selected to avoid interference from transplacentally transferred IgG maternal antibodies (the influence of breastfeeding is neglected, as it contains IgA which does not circulate in blood), and reduce the likelihood of two seasonal influenza virus infections [10,11]. Based on surveillance data of influenza A/H3N2 incidence in the Netherlands [19–21, 39–53], we determined the weeks in which the influenza-

like-illness exceeded 10 per 10.000 inhabitants, and selected two weeks either side of this epidemic season window (see **Supporting Dataset S1**); the two samples in the biobank collected before 1994 were not included, as they were drawn outside the epidemic season.

From this selection we retained those samples of which at least 150 μL serum was available to perform titrations, and omitted samples that appeared to be plasma or from individuals of whom we had already included a sample. The final sample selection included 72 sera.

Hemagglutination inhibition assay

We performed hemagglutination inhibition (HI) assays to screen the serum samples for influenza virus-specific antibodies, and to determine the antibody titers against a range of viruses for each influenza virus-positive serum. All serum samples were treated with cholera filtrate to remove nonspecific inhibitors of agglutination, and heat-inactivated at 56 ºC. Subsequently, the samples were tested with the HI assay using 1% turkey erythrocytes and 4 hemagglutinating units of virus, using the standard protocol [12,13]. A homologous ferret serum raised against a test antigen was used as a positive control for HI titrations. All sera were tested for non-specific agglutination by performing the incubation step in the absence of virus. The detection limit of the HI assay enables detection of titers 10 or higher, if there was no inhibition of agglutination, a titer of <10 was noted. Endpoint titers were determined for all titrations.

The serum samples were initially screened for the presence of influenza virus-specific antibodies, by titrating against the vaccine strain for the antigenic cluster that circulated in that season, see the rightmost column in **Supporting Dataset S1**. Of the 72 samples screened, 18 had a numeric titer (10 or higher) in the HI test against the screening virus.

These 18 influenza virus-positive samples were subsequently selected for further testing. We did not perform duplicates, in order for the limited serum volumes to be tested against a large panel of viruses. Each serum was tested with HI against a panel of 24 viruses: A/Wellington/5/1989 (WE/5/89); A/Guizhou/54/1989; A/Victoria/1/1989 (VI/1/89); A/Beijing/352/1989 (BE/352/89); A/Canberra/1/1991 (CA/1/91); A/Stockholm/7/92 (ST/7/92); A/Netherlands/938/1992 (NL/938/1992); A/Finland/220/1992 (FI/220/92); A/Shangdong/9/1993 (SD/9/93); A/Stockholm/20/1993 (ST/20/93); A/Johannesburg/33/1994 (JO/33/94); A/Finland/381/1995 (FI/381/95); A/Wuhan/359/1995 (WU/359/95); A/Sydney/5/1997 (SY/5/97); A/Hongkong/280/1997 (HK/280/97); A/Panama/2007/1999 (PM/2007/99); A/Netherlands/1/2002 (NL/1/02); A/Fujian/411/2002 (FU/411/02); A/California/7/2004 (CF/7/04); A/Wisconsin/67/2005 (WN/67/05); A/Brisbane/10/2007 (BR/10/07); A/Perth/16/2009 (PE/16/09); A/Victoria/361/2011 (VI/361/11); A/Netherlands/63/2011 (NL/63/11). The data for A/Guizhou/54/1989 were omitted, as the virus used had only 2, as opposed to the required 4, hemagglutinating units in the HI assay. Additionally, one serum that in the screening had a titer of 320, was subsequently found to be negative when tested against the full virus panel,

and was omitted from the study, leaving 17 influenza-virus positive human sera.

For comparative purposes, we tested the sera of 24 ferrets against the same panel of viruses with the HI assay. The ferret sera had been raised against the following antigens: A/Guizhou/54/1989 (GU/54/89); A/Netherlands/620/1989 (NL/620/89); A/Beijing/353/1989 (BE/353/89); A/Victoria/2/1990 (VI/2/90); A/Hongkong/34/1990 (HK/34/90); A/Beijing/32/1992 (2 sera: BE/32A/92, BE/32V/92); A/Guangdong/25/1993 (GD/25/93); A/Nanchang/933/1995 (NA/933/95); A/Netherlands/47/1995 (NL/47/95); A/Netherlands/218/1995 (NL/218/95); A/Sydney/5/1997 (SY/5A/97); A/Singapore/1/1996 (SP/1/96); A/Wyoming/3/2003 (WY/3/03); A/Netherlands/22/2003 (NL/22/03); A/Netherlands/88/2003 (NL/88/03); A/Newyork/55/2004 (NY/55/04); A/Wellington/1/2004 (WE/1/04); A/Hiroshima/052/2005 (HI/52/05); A/Uruguay/716/2007 (UR/716/07); A/Brisbane/10/2007 (BR/10/07); A/Netherlands/377/2008 (NL/377/08); A/Perth/16/2009 (PE/16/09); A/Victoria/210/2009 (VI/210/09).

Selection of sera and viruses

The 17 influenza-virus positive sera showed a dichotomy in their screening titers (two-sided t-test p=0.007), with a group of relatively high-responding individuals (n = 6, HI titer range against screening virus 240-3840), and low-responders (n = 11, HI titer range against screening virus 10-60). Firstly, the sera of the high-responders were analyzed. To reliably place a virus on an antigenic map, it needs at least two numeric titers in the HI table. Therefore,

only viruses that had at least two numeric values (HI titer 10 or higher) against these six sera were included, the subset of the HI table can be seen in **Supporting Dataset S4**. The same set of viruses was used to create the comparative antigenic map of ferret sera, where only sera were included that had at least two numeric values, and the highest titer for this subset of viruses had to be at least 240 (i.e. exclusion of sera BE/353/89, VI/2/90, and NY/55/04).

In a subsequent analysis two additional human sera were included that had a highest titer (against all titrated viruses) of at least 240: S10-1 and S10-2, to add sera from later clusters that showed expected reactivity patterns. Again viruses were included that had at least two numeric values, and for the comparative ferret antigenic map the same set of viruses was used, and only sera were included that had at least two numeric values and a highest titer for this subset of viruses of at least 240.

Antigenic maps

Antigenic cartography was performed using the HI tables containing titers of a set of sera against a panel of antigens. An antigenic map enables a quantitative and visual interpretation of the binding assay data, incorporating the multiple serum-virus relation measurements to enable reliable inferences about antigenic differences among pathogen strains [1]. An HI titer between a serum and virus is converted into a distance by calculating the difference between the log2(HI titer) of a virus-serum pair and the maximum log2(HI titer) of that serum against any virus. Therefore, a serum with a high HI titer

will have a shorter distance, and be placed more closely to that virus, than a serum with a lower HI titer. The viruses and sera are subsequently positioned in the map by minimizing the difference between the target distances and the distances in the antigenic map, using multidimensional scaling (500 dimensional annealing runs were performed as random restarts to avoid local optima). Titer differences are shown in two-fold dilutions (denoted as 1 antigenic unit), and so 2 grid units correspond to a four-fold dilution in the HI assay, 3 units to an eight-fold dilution, and so on.

When calculating an antigenic map, it is not necessary for the HI table to encompass the homologous sera matching the viruses tested, because any three different (serum) data points can triangulate a virus data point, as long as the titers are numeric. We have more than three ferret serum data points for all viruses, and thus the redundancy in the data will allow good coordination of the viruses and sera (as indicated by small symbol sizes in the figures) – and similarly for the human data.

Column basis

When calculating antigenic maps of ferret sera, a minimum column basis is typically used, because sera with only lower titers most likely are a result of the absence of homologous strains in the HI table. Thus, each serum is expected to be able to have a titer of at least 1280, even if the HI table only contains titers lower than that. However, for the children sera, the lower titers might have resulted from decay over time, instead of resulting from the absence of homologous strains. Because there is no reason to assume that

the children's titers should have been at least 1280 (as one can reasonably do for ferrets, based on many available data sets showing these kinds of titers, for a serum drawn two weeks post-infection), we decided to not use a minimum column basis, instead, the experimentally measured maximum titer of a serum was used to calculate the distance (the column basis). As an example, if the experimental maximum titer of a serum was 320, and used to calculate the difference this would lead to a calculated distance 2 units shorter than if the column basis would have been set to 1280 (as the subtraction was with log2(320/10)=5, instead of log2(1280/10)=7). For some analyses, we did set a minimum column basis of 1280. With this setting, if the maximum titer of that serum did not reach 1280, the maximum titer of a serum was still taken as 1280 (higher maxima do not get replaced).

Antibody landscapes

Whereas the human antigenic map was based on titers of all individuals, the 3rd dimension in the antibody landscape shows the individual titers of a single person. To construct the antibody landscapes, we altered the methodology described in Fonville *et al.* [6] to achieve a more robust fit by modeling the effects of the x and y antigenic coordinate variables independently, rather than in terms of their interaction as was previously described. Thus, the relationship between the measured titers z_i and the antigenic coordinate variables x and y, was modelled as follows for each antigen:

$$z_i = c_j + x_i\beta_{j_1} + y_i\beta_{j_2} + \varepsilon_i$$

where z_i is the measured titer for a given antigen i, x_i and y_i are the corresponding x and y coordinates of this measurement point, and c_j, β_{j_1} and

β_{j_2} are the regression coefficients used when determining the local fit at point p_j. As before, to calculate the estimated landscape height at point p_j, the regression coefficients c_j, β_{j_1} and β_{j_2} were resolved to minimize the weighted sum of squares of the errors ε_i across all antigens i. This local regression is performed independently for each individual point in the landscape. The weighting of the local regression was performed with smoothing parameter A set to 8 antigenic units, based on visual inspection of the interpolated fits.

When creating the antibody landscapes of the six human sera on the ferret-sera based antigenic map, instead of the human-sera based antigenic map, the error of the fit increased, as may be expected, from a root mean sum of squares across the six sera and all viruses of 0.96 to 1.18 antigenic units.

Supporting Tables:

Of the 17 patients that were found to have antibodies against influenza A/H3N2 in this study, 4 had underlying medical conditions (congenital kidney disease, bone formation disorder, neonatal cataract and recurrent wheezing), see **Table S1**.

Table S1. The frequencies for the various reasons for hospitalization and diagnostic tests of the 17 patients.

**Reason for hospitalization and diagnostic tests**	**N =**
Gastroenteritis infection	1
Follow up after HIV exposure (non-infected)	2
Rash	2
Follow up congenital kidney disease (non-autoimmune)	1
Respiratory infections	1
Anemia	1
Neonatal cataract	1
Screening after stay in tropics (not during influenza epidemics)	3
Bone formation disorder	1
Lymphadenopathy	2
Failure to thrive	1
Check vaccination response	1

Supporting Figure Legends:

Figure S1: The antigenic map of the six high-responding human sera shown in **Figure 2** with error lines. An error line displays the difference between the HI titer and the distance in the antigenic map, and one half is drawn as a line originating in the virus position, the other half from the serum. Thus, the distance between the ends of a pair of error lines represents the HI titer. Red lines indicate the amount by which the HI titer exceeded the map distance, blue lines indicate how much the map distance exceeded the measured HI titer.

Figure S2: A) The antigenic map of the six high-responding human sera made with a minimum column basis of 1280. B) The map shown in **Figure S2A** with procrustes arrows indicating the position of each serum and virus in the map of **Figure 2**.

Figure S3: A) The antigenic map of the ferret sera shown in **Figure 3** made with a minimum column basis of 1280. B) The map shown in **Figure S3A** with procrustes arrows indicating the position of each virus in the human map made with a minimum column basis, shown in **Figure S2A**.

Figure S4: A) The antigenic map based on a subset of six ferret sera (without minimum column basis). To allow fair comparison with the human sera map, we determined for each human serum a matching ferret serum (by calculating the differences between the logged HI titers of the human serum and each

ferret serum, and taking the ferret serum of which the sum of the absolute differences was lowest). Serum S00-2 was matched most closely to NL/88/03, but since this ferret serum was already selected, the second best-matching serum SY/5A/97 was added. B) The map shown in **Figure S4A** with procrustes lines to the human map shown in **Figure 2**. C) The map shown in **Figure S4A** with procrustes lines to the ferret map shown in **Figure 3A**.

Figure S5: Antibody landscapes of each of the six high-responding human sera (in blue), and a ferret serum (in red), made using the human antigenic map of the sera of the six human high-responders shown in **Figure 2**. When the titer of the human and ferret serum were identical, a grey color was used. Each human serum was matched to the ferret serum that had the maximum correlation value when comparing the ferret and human serum HI titers.

Supporting Dataset Legends:

Dataset S1: The season; corresponding weeks of influenza A/H3N2 incidence exceeding 10 cases of influenza-like-illness (ILI) per 10.000 inhabitants in the Netherlands [19–21,39–53]; the sample selection window based on 2 weeks around the epidemic interval; and the virus used to screen for influenza virus-positive samples.

Dataset S2: Sample information, screening titers and full hemagglutination inhibition (HI) data set of titers against 23 test viruses of all 17 influenza virus-positive children's sera.

Dataset S3: HI titers of 24 ferret sera to the 23 test viruses.

Dataset S4: The subset of the HI table used to calculate the antigenic map based on the high-responding human sera (**Figure 2**).

Dataset S5: The subset of the HI table used to calculate the antigenic map of ferret sera in **Figure 3** using the same subset of viruses as **Dataset S4**.

Dataset S6: The subset of the HI table used to calculate the antigenic map based on all human sera that had at least one titer of 240 or higher (**Figure 4A, B**).

Dataset S7: The subset of the HI table used to calculate the antigenic map of ferret sera in **Figure 4C, D** using the same subset of viruses as **Dataset S6**.

Supporting References:

39. Claas ECJ, de Jong JC, Bartelds AIM, Rimmelzwaan GF, van Wijngaarden JK, Osterhaus ADME. Influenza in het seizoen 1994/’95; vaccinsamenstelling voor het seizoen 1995/'96. Ned Tijdschr Geneeskd **1995**; 139:2154–8.
40. Claas ECJ, de Jong JC, Bartelds AIM, Rimmelzwaan GF, van Wijngaarden JK, Osterhaus ADME. Influenza in het seizoen 1995/’96; vaccinsamenstelling voor het seizoen 1996/'97. Ned Tijdschr Geneeskd **1996**; 140:2047–50.
41. Rimmelzwaan GF, de Jong JC, Bartelds AIM, Claas ECJ, van Wijngaarden JK, Osterhaus ADME. Influenza in het seizoen 1996/’97; vaccinsamenstelling voor het seizoen 1997/'98. Ned Tijdschr Geneeskd **1997**; 141:1743–7.
42. Claas ECJ, Bartelds AIM, Dorigo-Zetsma JW, Rimmelzwaan GF, de Jong JC, Osterhaus ADME. Het influenzaseizoen 1997/’98 en de vaccinsamenstelling voor 1998/'99. Ned Tijdschr Geneeskd **1998**; 142:2423–7.
43. Rimmelzwaan GF, de Jong JC, Bartelds AIM, Dorigo-Zetsma JW, Fouchier RAM, Osterhaus ADME. Het influenzaseizoen 1998/’99; vaccinsamenstelling voor 1999/2000. Ned Tijdschr Geneeskd **1999**; 143:2015–8.
44. Rimmelzwaan GF, de Jong JC, Bartelds AIM, Dorigo-Zetsma JW, Fouchier RAM, Osterhaus ADME. Het influenzaseizoen 1999/2000 en

de vaccinsamenstelling voor het seizoen 2000/’01. Ned Tijdschr Geneeskd **2000**; 144:1968–71.

45. de Jong JC, Rimmelzwaan GF, Bartelds AIM, Wilbrink B, Fouchier RAM, Osterhaus ADME. Het influenzaseizoen 2000/’01 en de vaccinsamenstelling voor het seizoen 2001/'02. Ned Tijdschr Geneeskd **2001**; 145:1945–50.

46. Rimmelzwaan GF, de Jong JC, Bartelds AIM, Wilbrink B, Fouchier RAM, Osterhaus ADME. Het influenzaseizoen 2001/’02 en de vaccinsamenstelling voor het seizoen 2002/'03. Ned Tijdschr Geneeskd **2002**; 146:1846–50.

47. Rimmelzwaan GF, de Jong JC, Donker GA, Meijer A, Fouchier RAM, Osterhaus ADME. Het influenzaseizoen 2005/’06 in Nederland en de vaccinsamenstelling voor het seizoen 2006/’07. Ned Tijdschr Geneeskd **2006**; 150:2209–14.

48. de Jong JC, Rimmelzwaan GF, Donker GA, Meijer A, Fouchier RAM, Osterhaus ADME. Het influenzaseizoen 2006/’07 in Nederland en de vaccinsamenstelling voor het seizoen 2007/’08. Ned Tijdschr Geneeskd **2007**; 151:2158–65.

49. Rimmelzwaan GF, de Jong JC, Donker GA, Meijer A, Fouchier RAM, Osterhaus ADME. Het influenzaseizoen 2007/’08 in Nederland: antigene variatie, resistentie tegen oseltamivir en de vaccinsamenstelling voor het seizoen 2008/'09. Ned Tijdschr Geneeskd **2008**; 152:2138–44.

50. de Jong JC, Rimmelzwaan GF, Donker GA, Meijer A, van der Hoek W, Osterhaus ADME. De Mexicaanse grieppandemie van 2009: een

overzicht met focus op Nederland. Nederlands Tijdschrift Medische Microbiologie **2011**; 19:6–12.

51. de Jong JC, Donker GA, Meijer A, van der Hoek W, Rimmelzwaan GF, Osterhaus ADME. Het influenzaseizoen 2010/2011 in Nederland: het nieuwe A (H1N1)-virus van 2009 blijft actief. Nederlands Tijdschrift Medische Microbiologie **2011**; 19:21–7.

52. de Jong JC, Meijer A, Donker GA, van der Hoek W, Rimmelzwaan GF, Osterhaus ADME. Het influenzaseizoen 2011/12 in Nederland. Een kleine epidemie gedomineerd door het A(H3N2)-virus. Nederlands Tijdschrift Medische Microbiologie **2012**; 20:142–8.

53. de Jong JC, Donker GA, Meijer A, et al. Het influenzaseizoen 2012/2013 in Nederland: een milde maar langdurige epidemie. Nederlands Tijdschrift Medische Microbiologie **2013**; 21:135–42.
