# Supplementary figures and images for: Antigenic Maps of Influenza A(H3N2) Produced With Human Antisera Obtained After Primary Infection
Source: J Infect Dis. 2015 Jul 3;213(1):31–8. doi: 10.1093/infdis/jiv367 (PMC4676547; doi:10.1093/infdis/jiv367)

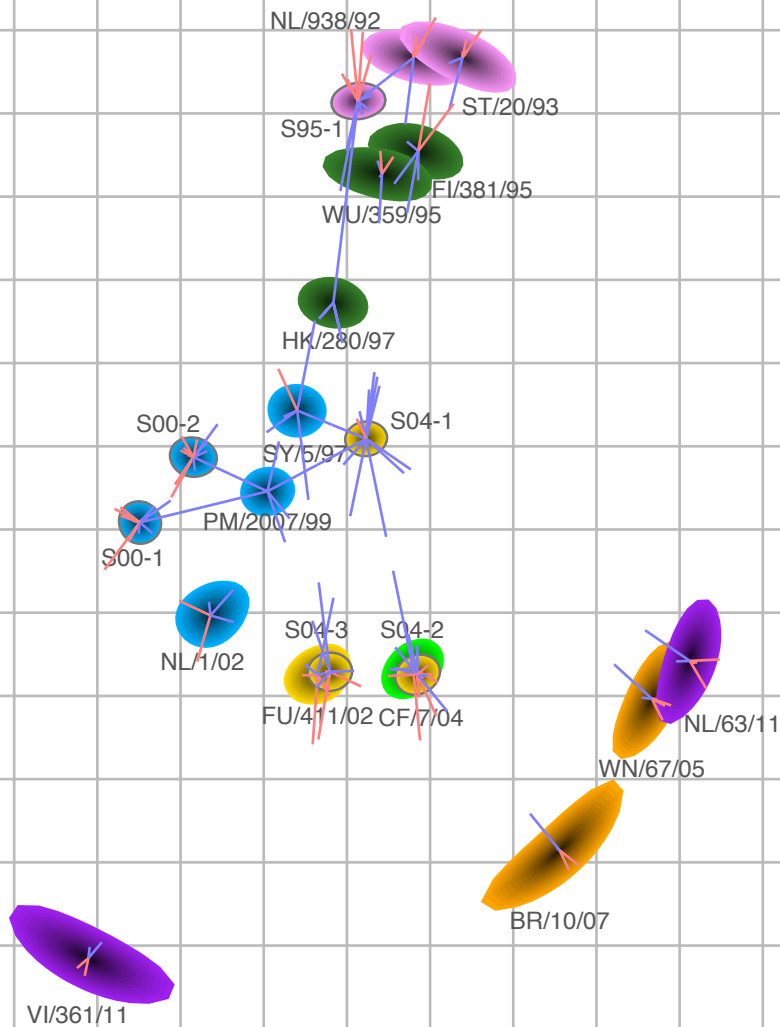

A

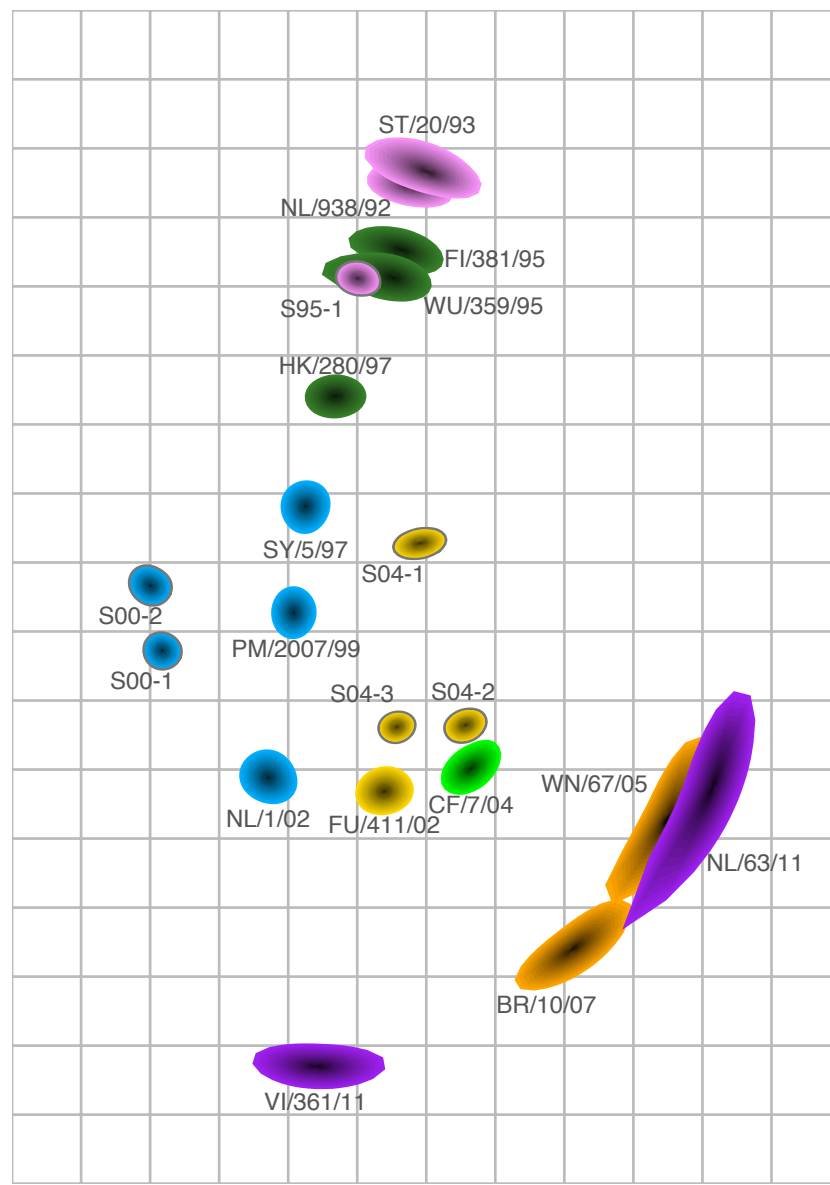

B

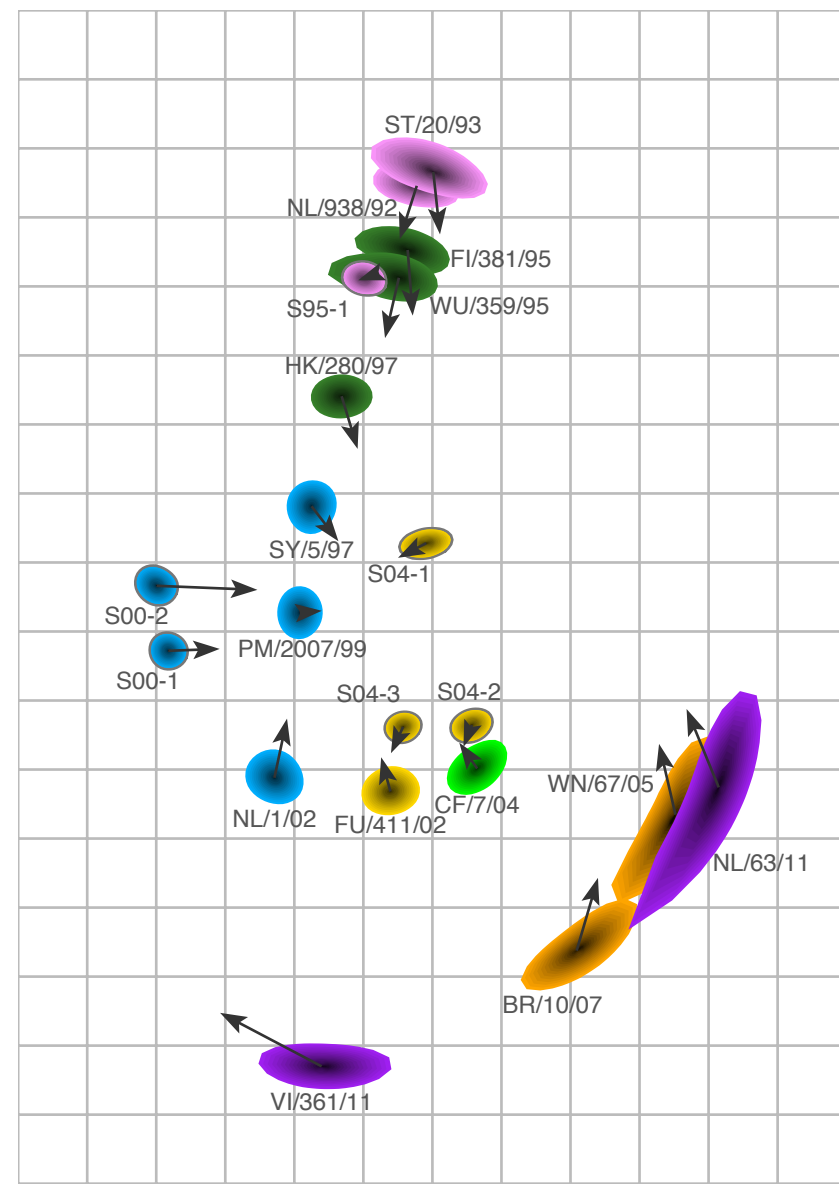

A

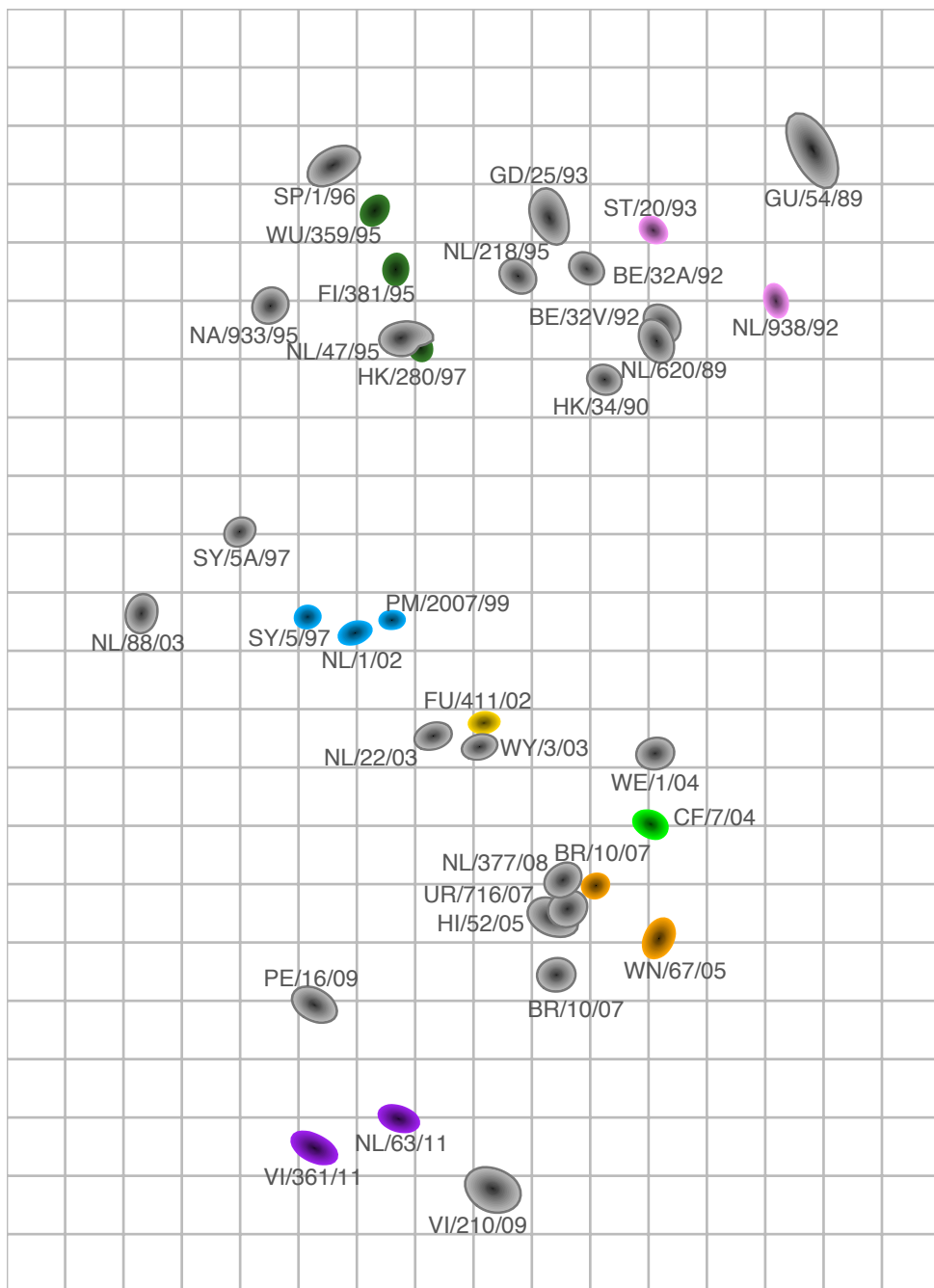

B

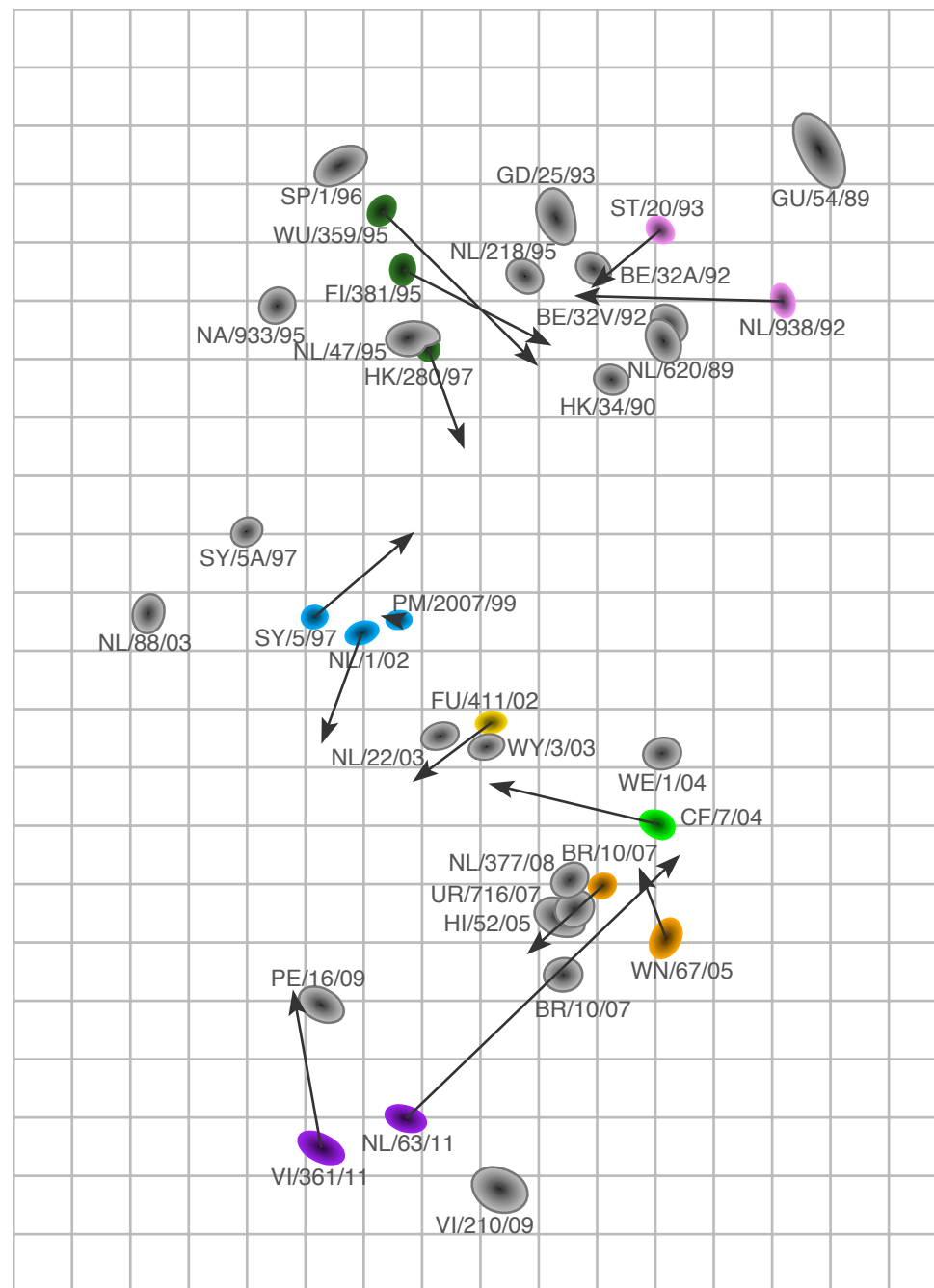

A

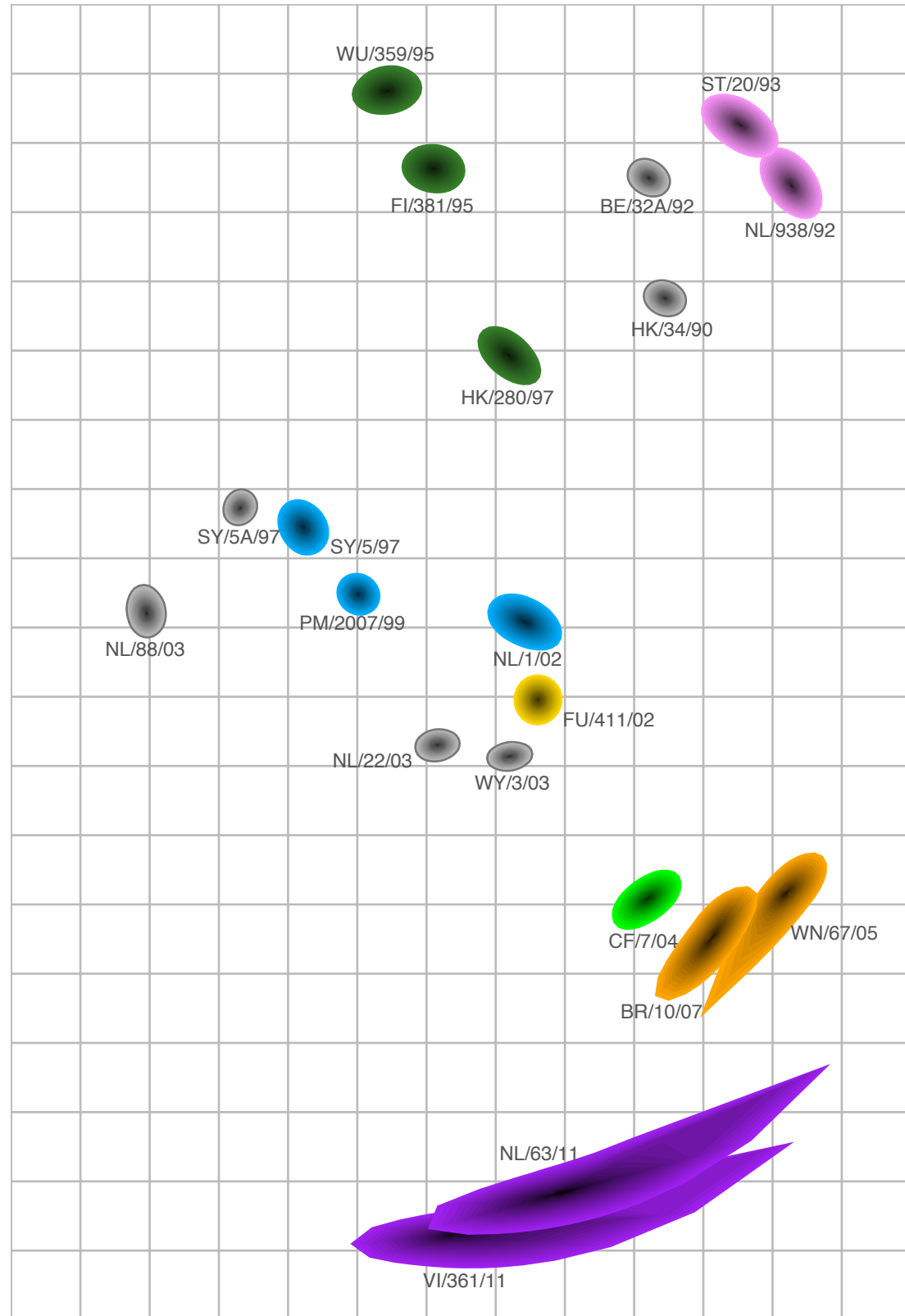

B

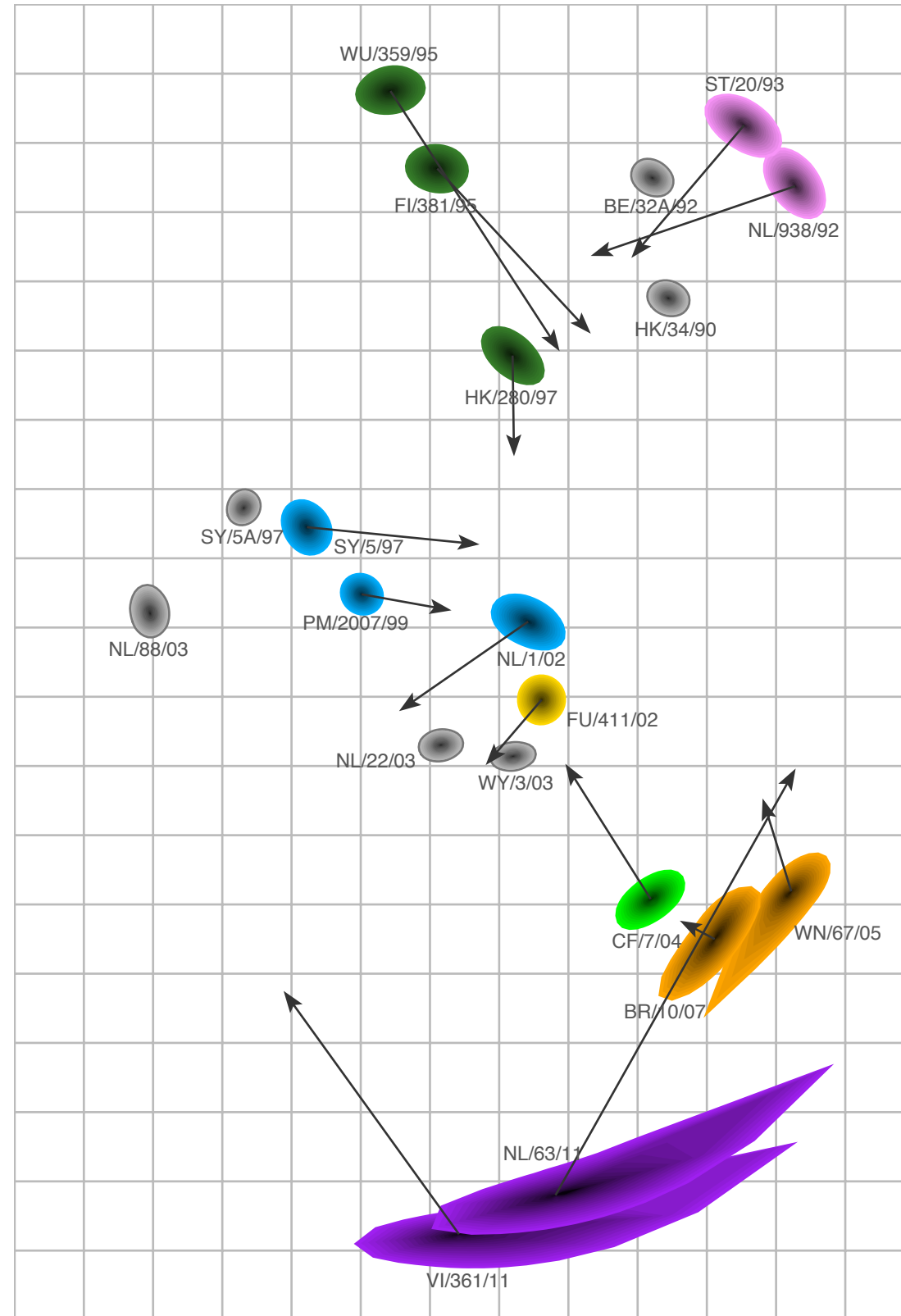

C

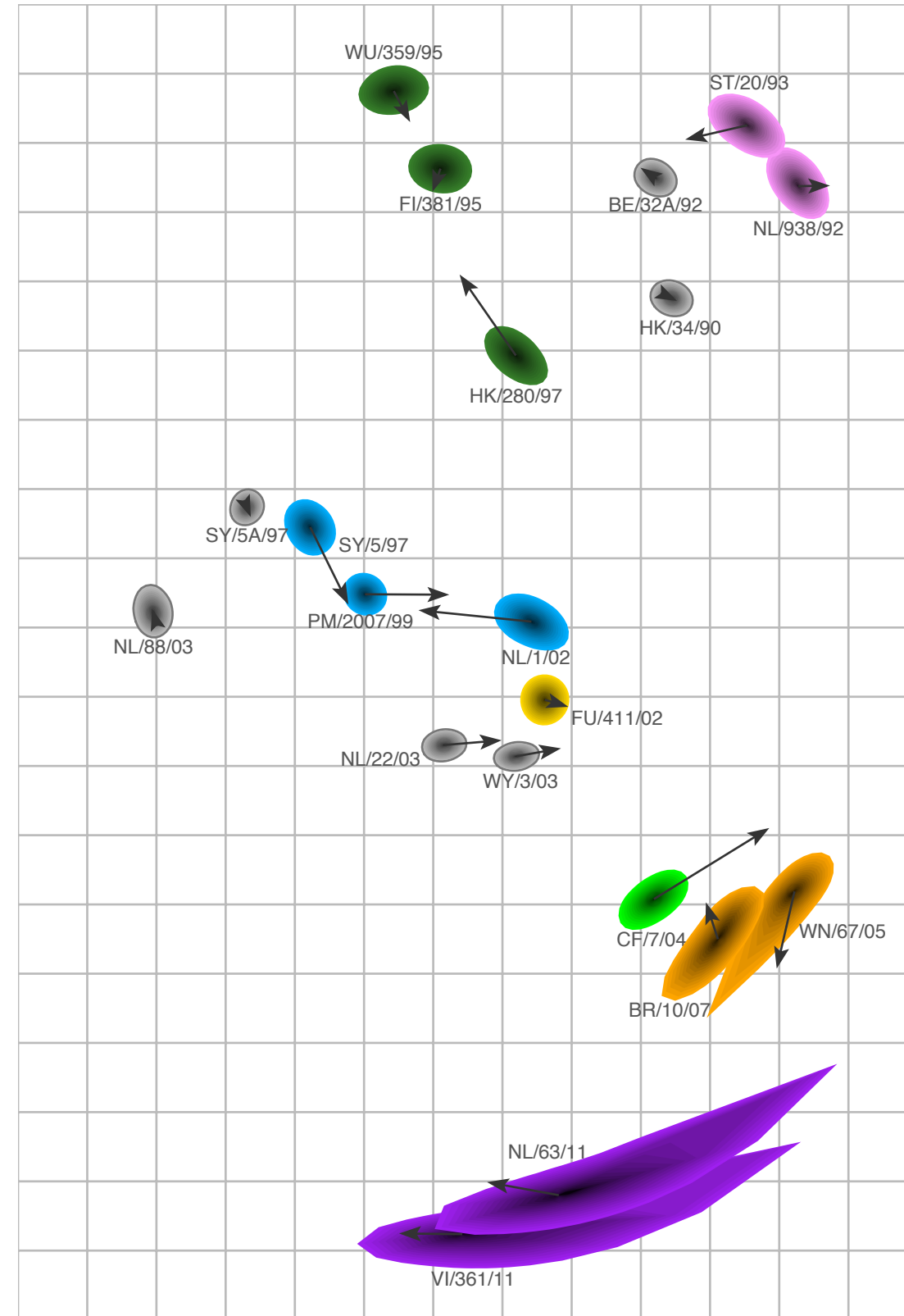

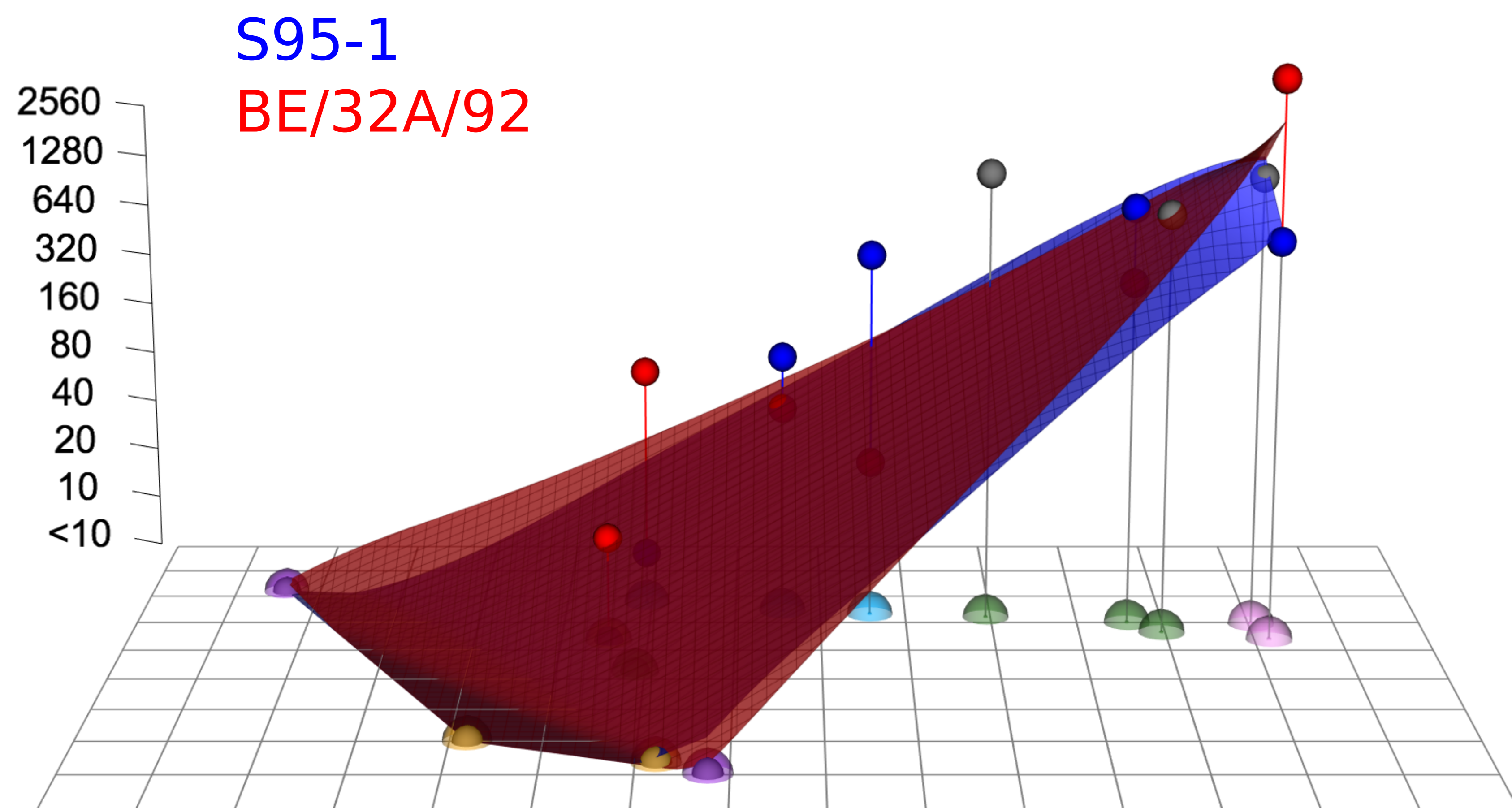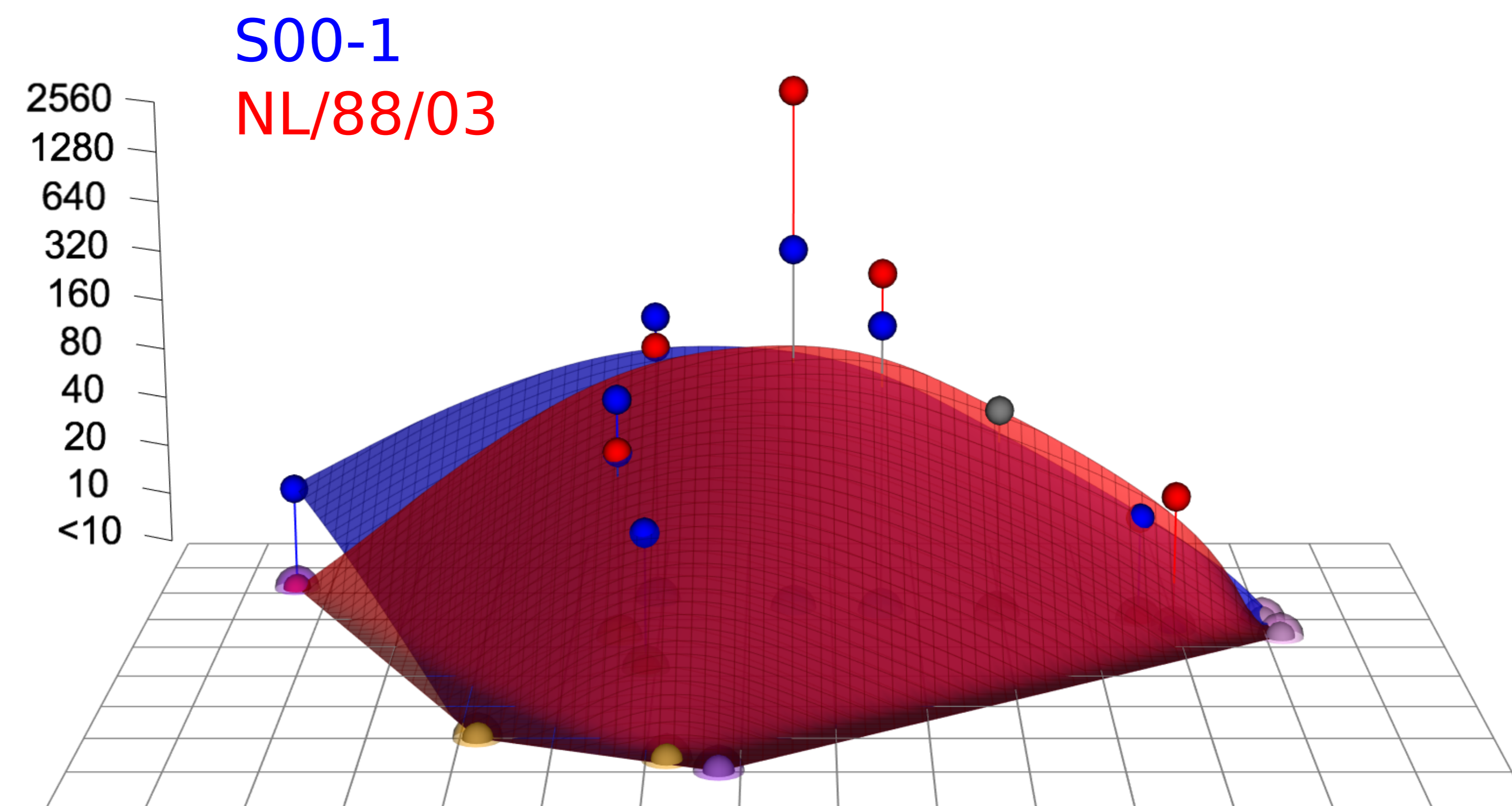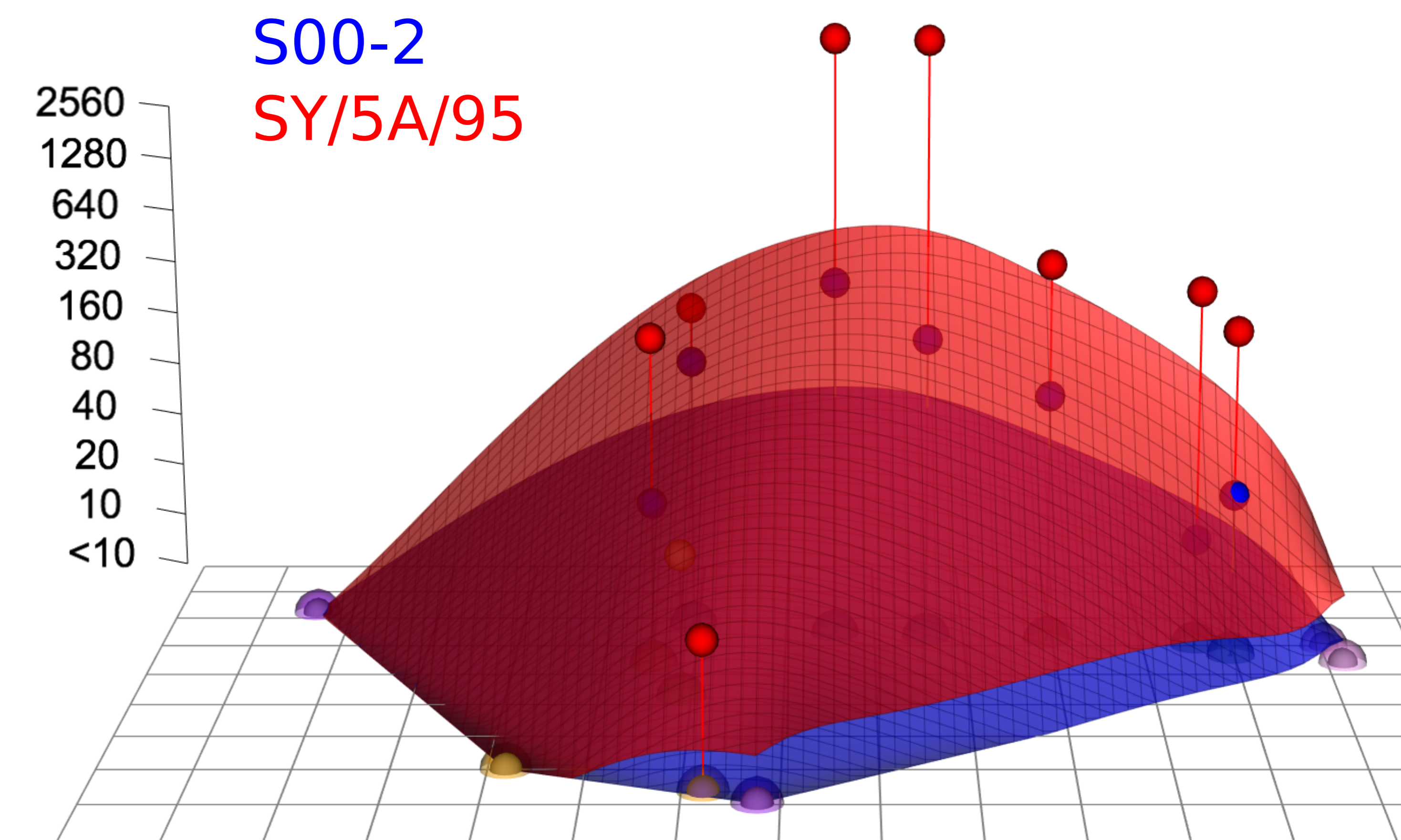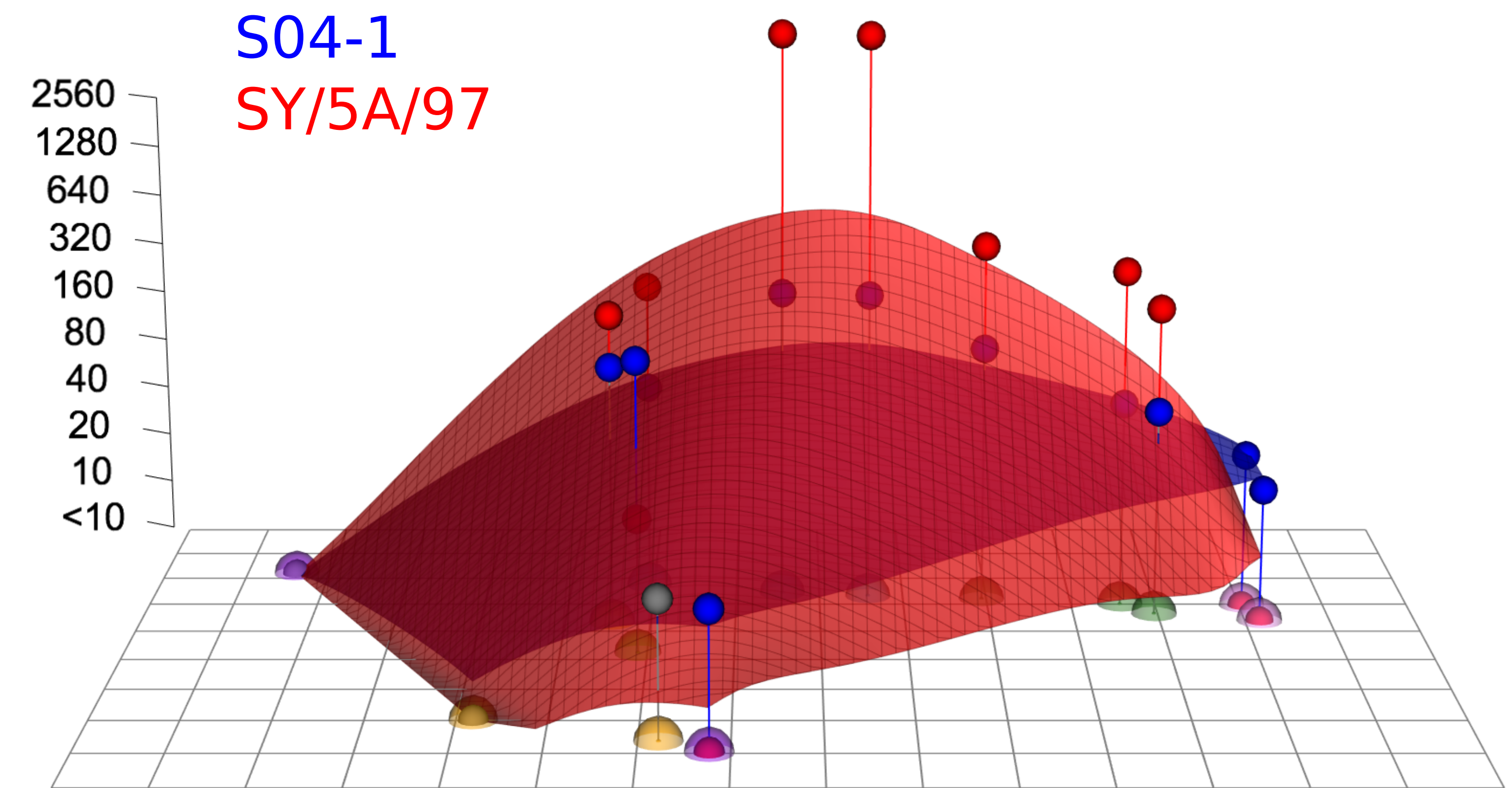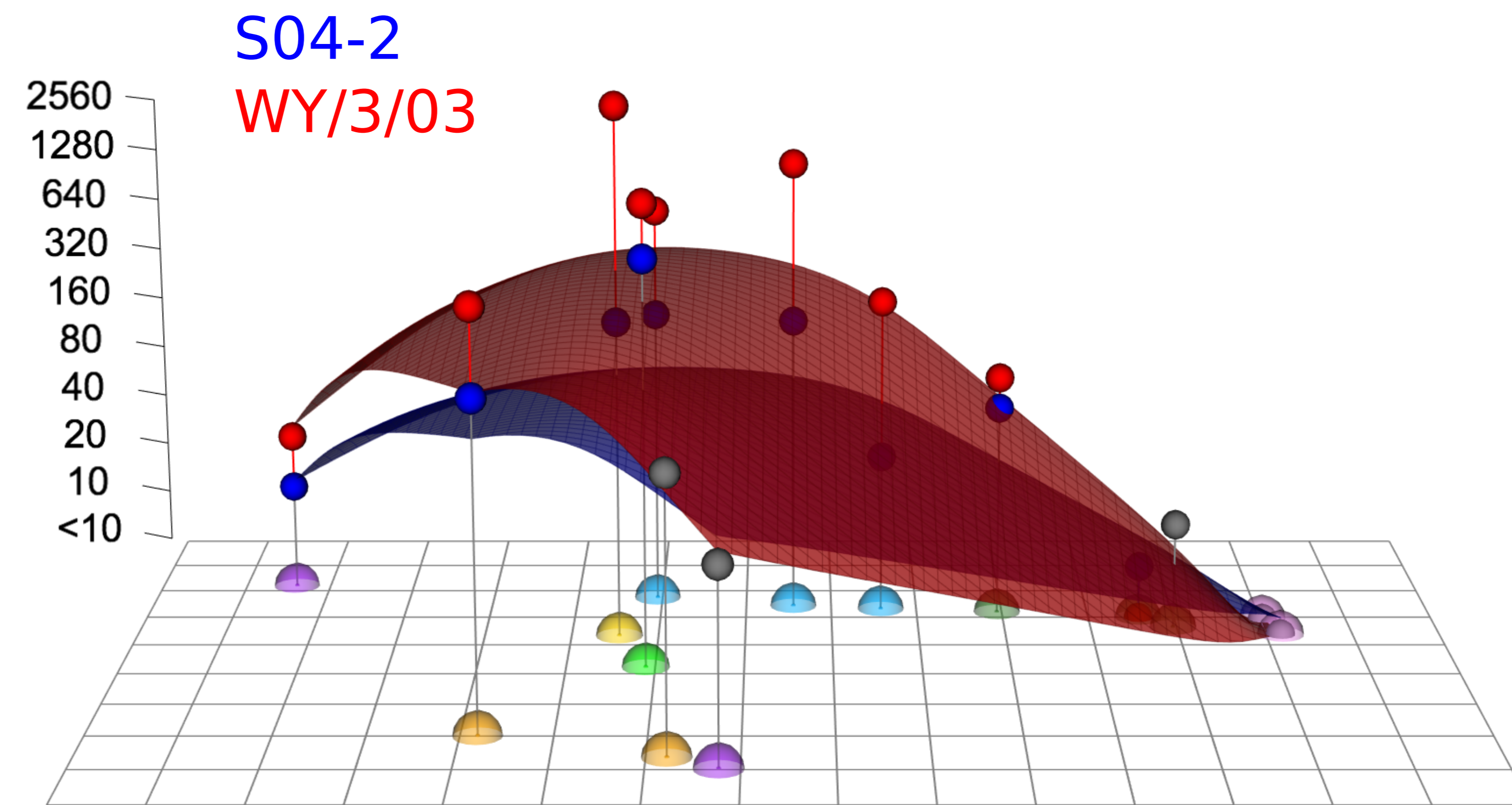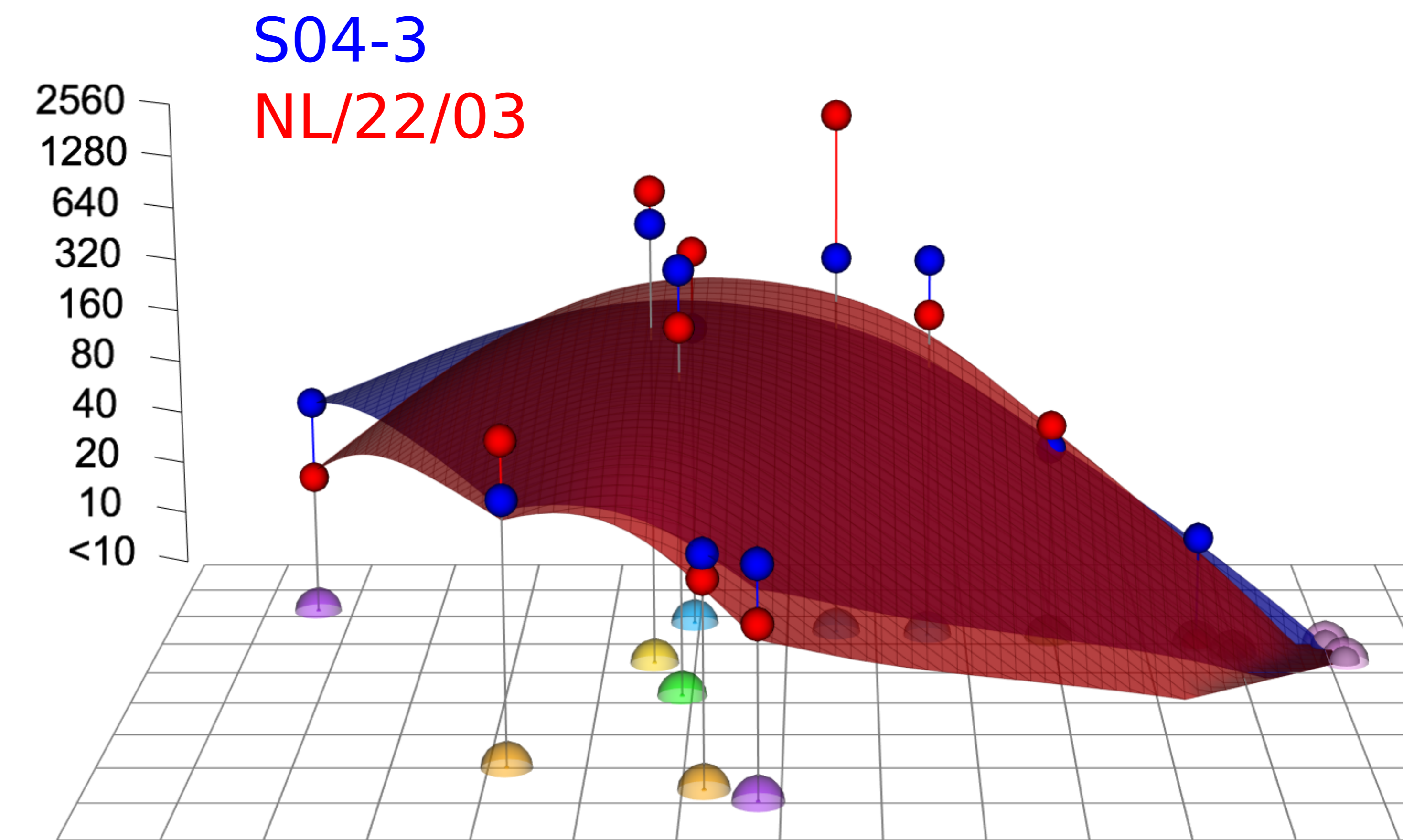

Supplement: Supplementary Data [file supp_jiv367_jiv367supp_figs.pdf]
